# Safety concerns in simultaneous use of prescription and 'over-the-counter’ medicines- results of patient survey in Estonia

**DOI:** 10.1186/2193-1801-3-143

**Published:** 2014-03-17

**Authors:** Maia Gavronski, Daisy Volmer

**Affiliations:** Department of Pharmacology, Centre of Excellence for Translational Medicine, Faculty of Medicine, University of Tartu, 19 Ravila Str., Tartu, 50411 Estonia; Department of Pharmacy, Faculty of Medicine, University of Tartu, 1 Nooruse Str., Tartu, 50411 Estonia

## Abstract

During the last decades, the share of population using prescription (Rx) medicines has considerably increased. With the wider introduction of self-medication and the use of over-the-counter (OTC) medicines, there is a real threat for drug-drug interactions between Rx and OTC medicines neither identified nor overseen by healthcare specialists.

The objectives of this survey were to ascertain how often, and for what conditions OTC and Rx medicines are used simultaneously, and to discuss possible health hazards connected with the concomitant use of these medicines.

This survey was designed as a descriptive, cross-sectional questionnaire-based interview which was conducted amongst pharmacy customers and patients in urban and rural areas of Estonia in between 2010–2012.

In total, 712 respondents participated in the survey. Of those, 50.4% reported concomitant use of Rx and OTC medicines during the survey. The simultaneous use of Rx and OTC medicines increased with age and the number of chronic diseases (both p < 0.001). Of chronic patients, 37.1% used Rx and OTC medicines on a daily basis over a four-week period before the survey, and considering reported chronic diseases and the most widespread OTC medicines, they could encounter drug-drug interactions between Rx medicines (e.g. antihypertensives, anti-inflammatory medicines) and OTC medicines (e.g. paracetamol, NSAID-s, herbal medicines and adsorbents).

The present survey revealed frequent concomitant use of Rx and OTC medicines. Especially vulnerable are chronic and elderly patients. In the future, both patients and healthcare specialists should pay more attention to possible drug-drug interactions of Rx and OTC medicines.

## Introduction

The use of over-the-counter (OTC) medicines has increased significantly in recent years (Amoako et al. 2003; Goh et al. 2009), but despite the positive aspects, such as improved availability of medicines, decreased number of physicians’ visits for minor illnesses and self-limiting conditions, and the increase of patients involvement in their treatment, patients do not fully comprehend the risks accompanying self-treatment, such as prolonged use, wrong dosage, side effects, and possible drug-drug interactions (Eickhoff et al. 2012). Patients consider OTC medicines to be safe (Ngo et al. 2010; Wawruch et al. 2013) and only regard their positive effects; therefore, they often ignore patient information leaflets and base their use of medicines on prior experience (Cullen et al. 2006; Hanna and Hughes 2011; Hughes et al. 2002; Wirtz et al. 2009).

Previous studies have confirmed that among the other age groups, OTC medicines are purchased and more often used by elderly patients (Sihvo et al. 2000) who frequently have multiple comorbid diseases, and therefore use polypharmacy. As a result, drug-drug interactions between OTC and prescription medicines (Rx) may occur. While patients are aware of possible drug-drug interactions between Rx medicines, patient knowledge regarding interactions between OTC and Rx medicines has not been well studied, although the occurrence of the aforementioned interactions has been described as frequent (Olesen et al. 2013; Sihvo et al. 2000).

Olesen et al. found that in Denmark, 50% of elderly patients taking OTC medicines were exposed to potential interactions (Olesen et al. 2013); and Sihvo et al. described that four percent of OTC users had taken drug combinations with the potential for clinically significant interactions (xSihvo et al. 2000). Based on this knowledge, it is necessary to study the frequency of and possible risks with the concomitant use of OTC and Rx medicines in more detail.

The objectives of this research were:

- to ascertain how often and for what conditions OTC and Rx medicines are used simultaneously, according to self-assessment of Estonian residents, and
- to discuss possible health hazards connected with concomitant use of OTC and Rx medicines.

## Materials and methods

### Setting and survey sample

A questionnaire-based interview was conducted among pharmacy customers and patients at five community pharmacies and six general practitioner (GP) centres in urban areas (Tallinn, Tartu and Narva) and in rural areas (islands Saaremaa and Ruhnu) of Estonia in between 2010–2012. Depending on the region, the survey period lasted four to six weeks. In the rural areas with lower population density the study period was prolonged to six weeks for reaching respective number of participants.

The interviews were performed on working days from 11:00 AM–17:00 PM. The vast majority of pharmacy customers or patients aged between 15–85 years, purchasing Rx or OTC medicine at a community pharmacy or approaching to registration desk at a GP centre were invited to participate in survey. In all regions, the respondents were interviewed by pharmacy students (in all regions one pharmacy student per community pharmacy and one at GP centre) who were previously trained to conduct interviews and respond to possible questions asked by customers or patients. Respondents were informed about the aims of the survey and their participation was voluntary. Definitions of Rx and OTC medicines and chronic conditions were provided only to those respondents who required further guidance. Interviewing was performed in a separate room or isolated section in sales area at community pharmacy or registration area at GP centre. The average interviewing time was 10 minutes.

Face-to-face interviews in the form a pre-structured questionnaire were selected to obtain more reliable information. Trial period of the questionnaire demonstrated insufficient knowledge of lay people about the names of active components in OTC medicines, and a self-administered questionnaire would have resulted in incomplete replies or in the misinterpretation of items that has also been experienced in earlier international research (Cuzzolin and Benoni 2010).

The present research did not use identifiable human material or data. The survey conforms to the Declaration of Helsinki and the ethical standards of Estonia. The researchers have fulfilled the Data Protection Act of Estonia.

### Survey instrument

Survey instrument was developed by using the survey models applied in our previous research on community pharmacy services in Estonia, Estonian Health Interview Survey, and on an Italian survey about the safety of OTC medicines (Cuzzolin and Benoni 2010; Estonian Health Interview Survey 2013; Volmer et al. 2009). The content validity of the survey instrument was assessed by a panel of researchers in social pharmacy, primary health care, and pharmacy practice. The survey instrument was pilot tested for face validity among a convenience sample of ten pharmacy customers and patients. Based on the feedback, minor changes were made to the wording of the items.

Overall, the structured survey instrument comprised of 17 mainly multiple-choice items related to the respondents’ demographic background (age, gender, education, region, and data collection location); their knowledge about the use of OTC medicines for the treatment of more common minor ailments (gastrointestinal tract and respiratory tract disorders, minor skin complaints and allergy, recurrent headache and other aches and pain), and about concomitant use of OTC and Rx medicines in the case of chronic diseases. To identify the simultaneous use of Rx and OTC medicines, respondents were asked to indicate the use of Rx and/or OTC medicines at the time of the survey and the frequency of concomitant use of described medicines one month prior to the survey.

In addition, respondents’ attitudes toward community pharmacies as information sources about OTC medicines were asked. The analyses outlined in this manuscript relates only to the concomitant use of Rx and OTC medicines.

In the current survey OTC medicines were defined as non-prescription medicines, herbal medicines and dietary supplements not licensed as prescription medicines in Estonia. The respective information was received from the register of authorised medicinal products at the State Agency of Medicines.

Rx medicines were identified by means of the names of chronic diseases where the use of Rx medicines is consistent, as INN (International Nonproprietary Name) and the trade names of Rx medicines were less familiar to patients.

Chronic disease was defined as a human health condition or disease that is persistent for longer than three months and cannot be prevented or treated by medicines.

In this survey people who were interviewed at community pharmacy were defined as pharmacy customers and those interviewed at GP centre as patients.

A copy of the survey instrument can be obtained by contacting the corresponding author.

### Statistical analyses

For statistical analysis, IBM SPSS Statistics v. 19.0.0.2 was used. Cross tabulations and Chi Square tests and Cramer’s V were employed to evaluate statistical correlations between demographic data of the respondents and their replies. Statistical significance was set at the level p < 0.05.

## Results

### Demographics

In total, 712 individuals were interviewed during the survey period. The number of patients/customers who refused to participate in the study was not recorded. In the survey, age distribution of the population was almost equal and corresponded to the age groups of general population in Estonia. However, 2/3 of the respondents were females and from urban regions. More than half of the respondents reported chronic disease with regular use of Rx medicines and simultaneous use of OTC medicines for treatment of minor ailments (Table 1).

**Table 1:** **Demographic characteristics of the respondents (n = 712)**

Demographic indicators	n	%
*Age (years)*		
15–19	57	8.0
20–29	129	18.1
30–39	118	16.6
40–49	138	19.4
50–59	84	11.8
60–69	96	13.5
70–85	90	12.6
*Gender*		
Female	518	72.9
Male	194	27.1
*Education*		
Primary/secondary school	327	45.9
Secondary school education with specialisation	220	30.9
University degree	165	23.2
*Region*		
Urban area (Tartu, Tallinn,Narva)	533	74.9
Rural area (Saaremaa and Ruhnu)	179	25.1
*Survey location*		
Pharmacy	363	51.0
GP centre	349	49.0
*Chronic disease*		
Incidence of chronic disease with concomitant use of Rx and OTC medicines	448	63.0
Incidence of chronic disease was not indicated	264	37.0

### Chronic diseases and the use of Rx and OTC medicines

**Figure 1:**
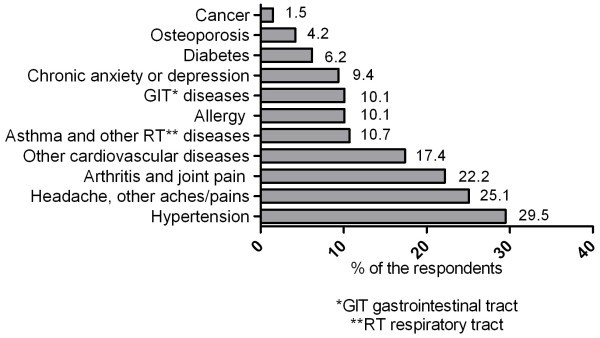
**Incidence of chronic diseases when the concurrent use of Rx and OTC medicines was indicated.**

For identification of what Rx medicines respondents have used concomitantly with OTC medicines survey participants were asked to indicate their chronic disease(s) require regular use of Rx medicines. More frequently listed diseases and conditions included hypertension (n = 210), recurrent headache and other aches and pain (n = 179), arthritis and joint pain (n = 158), and other cardiovascular diseases (n = 124) (Figure 1).

### Concurrent use of OTC and Rx medicines

**Figure 2:**
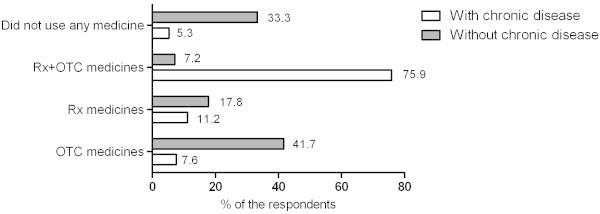
**The use of Rx and OTC medicines at the time of survey among Estonian residents with chronic disease and without chronic disease.**

During the period when the survey was conducted, 20.2% (n = 144) of the respondents used only OTC medicines, 13.6% (n = 97) used only Rx medicines, and 50.4% (n = 359) reported simultaneous use of both medicines (Figure 2).

During the four weeks prior to the survey, 24.6% (n = 175) of survey participants in total and 37.1% (n = 264) of those suffered from at least one chronic disease used Rx and OTC medicines simultaneously on a daily basis. In different age groups, concomitant daily use of Rx and OTC medicines increased from 12.5% in the 15–19 years group to 65.0% in the 70+ years group (p < 0.001; Cramer’s V 0.23). The use of Rx and OTC medicine was more frequent (p < 0.001; Cramer’s V 0.25) with patients with an increased number (3–4) of chronic diseases. In the comparison of urban and rural regions, the concurrent daily use of Rx and OTC medicines was slightly higher in rural areas (p < 0.001; Cramer’s V 0.17).

**Figure 3:**
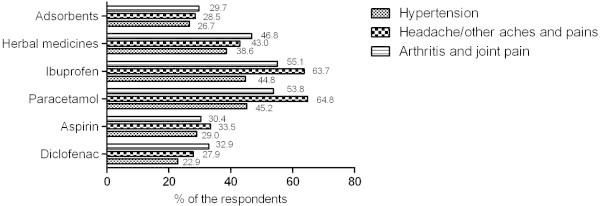
**At the time of survey more frequently used OTC medicines by chronic patients with regular use of Rx medicines.**

The use of OTC medicines was studied in more detail among those respondents who indicated chronic disease and simultaneous use of Rx and OTC medicines. Figure 3 illustrates more frequently used OTC medicines in the case of more prevalent chronic diseases or conditions where the use of Rx medicines is mostly required. The reported OTC medicines were mostly used for treatment of minor complaints. While the top consisted mostly of widely used OTC medicines containing NSAIDs (non-steroidal anti-inflammatory drugs) and paracetamol, traditional treatments (herbal medicines and adsorbents - charcoal) were also popular (Figure 3). Last mentioned OTC medicines were frequently used for relieving minor respiratory and gastrointestinal tract complaints.

It was common to use 2–3 different OTC medicines to cure one type of minor illnesses. However, the survey instrument did not allow to determine, whether the different medicines were used simultaneously or not. When respondents already showed the symptoms of a minor illness, only a few of them used vitamin and/or mineral preparations or probiotics to support the body during the convalescence period, or to prevent possible minor illnesses in the future.

## Discussion

This is the first survey among Estonian residents describing the concomitant use of Rx and OTC medicines. Several international surveys have previously described possible risks in the use of Rx or OTC medicines (Olesen et al. 2013; Sihvo et al. 2000). However, there has been little interest in the description of clinical hazards that could manifest in the interactions of Rx and OTC medicines (Bond and Hannaford 2003).

### Factors influencing identification of drug interactions

The results of the present survey do not describe the impact of pharmaceutical policy to the identification and evaluation of drug interactions. However, this knowledge would serve as clarification to the reasons why drug interactions occur and this process is not controlled by healthcare specialists.

Estonia has been described as a good example of the implementation of the Electronic Health Record (EHR). As a part of this system, digital prescription was introduced in 2010. Currently, GPs can follow the dispensing of prescribed medicines but still miss information about OTC medicines purchased independently by the patient. Drug communication that takes place at the community pharmacy is mainly connected with currently dispensed Rx or OTC medicines as pharmacists have no access to patients’ medical records. On the other hand, community pharmacists could be well positioned health care specialists, who evaluate the use of both Rx and OTC medicines, as in Estonia the sale of OTC medicines is limited to pharmacies. In the current situation, it depends on the patients whether they inform their GP about the use of OTC medicines, and the pharmacist about Rx medicines, and whether they receive evidence-based information concerning possible interactions (Estonian EHR Case Study 2013; Implementation of digital prescriptions in Estonia 2013).

Leemans et al. have stressed the accessibility of medical records by pharmacists as an important factor in the elimination of prescription errors, including the identification of clinically important drug-drug interactions (Leemans et al. 2003). However, other surveys describe the situation where drug-drug interactions are a much less discussed topic by both patients and community pharmacists compared to advice given about medicine administration and dosage (Cullen et al. 2006; Cuzzolin and Benoni 2010; Hanna and Hughes 2011; Hughes et al. 2002; Olesen et al. 2013; Sihvo et al. 2000; Volmer et al. 2009; Wirtz et al. 2009).

### Use and interactions of Rx and OTC medicines

This survey demonstrates the frequent use of Rx and OTC medicines, and the results are in accordance with previous research in Estonia. In between 1996–2006, the use of Rx medicines increased by 61% (30.9% in 1996 to 49.8% in 2006), and the number of patients using two or more prescription medicines simultaneously has doubled (Volmer et al. 2012). Another survey stressed that ¼ of the population in Estonia is using OTC medicines on a daily or weekly basis and 2/3 has made the decision independently without consulting a healthcare specialist (Kiivet 2009).

Similarly to Rx medicines, clinically significant pharmacokinetic and pharmacodynamic interactions might occur between OTC and Rx medicines (Strandell and Wahlin 2011). In this survey, ibuprofen and paracetamol were the most frequently used OTC medicines, followed by acetylsalicylic acid, and diclofenac (in doses 12.5 mg, or locally) that the respondents had taken to alleviate headaches, joint pain, and muscular pain. Nearly half of ibuprofen users indicated hypertension as a chronic comorbid condition; also, almost one-fourth of the respondents who used acetylsalicylic acid and diclofenac suffered from high blood pressure. The use of NSAIDs concomitantly with hypertension medicines has also been noted by other authors (Goh et al. 2009; Sihvo et al. 2000). It should be borne in mind that NSAIDs influence the effectiveness of antihypertensive medicines, particularly β-blockers, but also ACE inhibitors (Angiotensin-converting-enzyme inhibitor), and ARBs (Angiotensin receptor blocker), and the effect of loop diuretics could be weakened (Aljadhey et al. 2012; Fournier et al. 2012). In addition, long-term simultaneous use of ACE inhibitors and ARBs with NSAIDs has been described to cause renal impairment (Lapi et al. 2013). According to these findings, it is necessary to check patients’ blood pressure and renal function on a regular basis.

Arthritis with pain syndrome was mentioned as another chronic disease. Previous studies that have revealed that for pain alleviation, patients quite often disregard the amount of maximum doses – these are exceeded, or several different NSAIDs are used simultaneously, which significantly increases the risk of gastrointestinal and other adverse effects (Cavagna et al. 2013;x Hersh et al. 2007). Hence, to avoid the exceeding of maximum daily dosages, healthcare specialists should provide more extensive information about drug-drug interactions and side effects.

As a particular finding of this survey, adsorbents (charcoal tablets) and herbal medicines were frequently used by patients with hypertension, arthritis, headaches, and other type of pain to treat minor illnesses or complains. Charcoal tablets have traditionally been used to relieve gastrointestinal tract discomfort (e.g. flatulence, diarrhea), and in the case of minor intoxications (AACT and EAPCCT 1999; Bond 2002; Hall et al. 1981). The present survey demonstrated that this tradition is still followed by many chronic patients who perhaps due to ignorance affect the absorption of other medicines, making it a problem that has already been described in earlier research (Ibezim et al. 1999).

In Estonia, herbal medicines have been reported as frequently used preparations to treat the cold and flu (Raal et al. 2013). The present survey revealed similar results as chronic patients used herbal medicines mostly for the treatment of minor respiratory tract ailments. Although our survey did not specify in detail what herbal medicines the respondents used, it should be mentioned that some herbal medicines (St John’s wort, etc.) may have a significant effect on the enzymatic activity of liver cytochromes and therefore influence the effectiveness of prescription medicines (Williamson 2003).

### Limitations

This survey did not allow to determine possible interactions of particular Rx medicines with OTC medicines. The pilot period of the survey demonstrated insufficient knowledge of lay people about active ingredient names and product names of OTC medicines. Consequently, instead of using INN (International Nonproprietary Name) and the trade names of Rx medicines, we decided to use the names of chronic diseases people are more familiar with to determine possible concomitant use of Rx and OTC medicines.

## Conclusion

The present survey highlighted the frequent use of Rx and OTC medicines concurrently. OTC medicines have been mostly used for self-treatment of minor illnesses. Patients who self-administer OTC medicines and have chronic diseases what require regular use of Rx medicines may involuntarily influence the effects of used medicines. Therefore, patients need to be constantly advised by healthcare professionals.

In the light of the current pharmaceutical policy in Estonia, the quality of guidance can be improved if both GPs and community pharmacists had access to patients’ overall medical record, which includes the history of using both OTC and Rx medicines. It may be argued that the access to a complete medication record is especially important for elderly patients, because side effects and drug-drug interactions manifest often among this group.
